# Correction: Removal of metals and inorganics from rendered fat using polyamine-modified cellulose nanocrystals

**DOI:** 10.1039/d3su90029k

**Published:** 2023-08-10

**Authors:** Ezequiel Vidal, Frank Alexis, José M. Camiña, Carlos D. Garcia, Daniel C. Whitehead

**Affiliations:** a Department of Chemistry, Clemson University 211 S. Palmetto Blvd, Hunter Hall Clemson SC 29634 USA cdgarci@clemson.edu +1 864 656 3128; b Departamento de Ingeniería Química, Colegio de Ciencias e Ingeniería, Universidad San Francisco de Quito (USFQ) Quito 170901 Ecuador; c Facultad de Ciencias Exactas y Naturales, Universidad Nacional de La Pampa La Pampa Argentina

## Abstract

Correction for ‘Removal of metals and inorganics from rendered fat using polyamine-modified cellulose nanocrystals’ by Ezequiel Vidal *et al.*, *RSC Sustain.*, 2023, https://doi.org/10.1039/D3SU00116D.

The authors regret that, in the original article, the details for author affiliation *b* were incorrect. The correct details are as follows:

Departamento de Ingeniería Química

Colegio de Ciencias e Ingeniería

Universidad San Francisco de Quito (USFQ)

Quito 170901, Ecuador

The full and correct list of author affiliations is given in this document.

The Royal Society of Chemistry apologises for these errors and any consequent inconvenience to authors and readers.

